# Beyond the bones: Extraskeletal osteosarcoma of the thigh

**DOI:** 10.1016/j.radcr.2023.03.009

**Published:** 2023-04-06

**Authors:** Bader Abou Shaar, Ghassan Awad El-Karim, Abdul Rahman Alsaied, Nadeem Almalki, Nader Ashraf, Ameera Almalki, Rishi Duggal, Sohaib Munir

**Affiliations:** a College of Medicine, Alfaisal University, Riyadh, Saudi Arabia; b Department of Diagnostic Imaging, Bluewater Health, Sarnia, ON, Canada; c Department of Medical Imaging, Juravinski Hospital, Hamilton, ON, Canada; d University of Toronto, Faculty of Medicine, Toronto, ON, Canada; e Faculty of Science, Western University, London, ON, Canada; f Faculty of Math, University of Waterloo, Waterloo, ON, Canada; g Department of Medical Imaging, Western University, London, ON, Canada

**Keywords:** Extraskeletal osteosarcoma, Osteogenic sarcoma, Soft tissue sarcoma, Osteoid matrix, Oncologic radiology, Chemotherapy

## Abstract

Extraskeletal osteosarcoma (ESOS) is a rare malignant mesenchymal soft tissue tumor that usually arises in the lower extremities. It is typically a high-grade malignancy that represents only around 1%-2% of all soft tissue sarcomas and 2%-4% of all osteosarcomas. In this report, we describe a case of a 67-year-old female who presented with a 4-day history of a painless lump in her posterior right thigh. Workup utilizing different imaging modalities yielded a diagnosis of ESOS. The radiologic features of ESOS, as well as the current treatment paradigm and prognosis of this rare malignancy, will be discussed based on a review of the literature.

## Introduction

Extraskeletal osteosarcoma (ESOS) is a high-grade malignant soft tissue tumor of mesenchymal origin. It characteristically synthesizes bone, osteoid, or chondroid material without directly attaching to the underlying periosteum or bone [1]. ESOS is an extremely rare malignancy, representing only around 1%-2% of all soft tissue sarcomas and 2%-4% of all osteosarcomas [2,3]. Although it was first described in 1941, to date, there are approximately only 390 cases reported in the literature [4], [5], [6], [7], [8], [9]. ESOS is mostly found adjacent to the long bones of the lower extremities, with a predilection for the deep tissues of the thigh, followed by the buttocks and trunk [2,10,11]. In this report, we describe a case of a 67-year-old female with ESOS in the posteromedial compartment of the right thigh. Despite the increase in awareness and knowledge about this rare tumor in the literature, there is a limited understanding of the clinical behavior and therefore consensus on therapeutic approach is lacking. The radiologic features of ESOS, as well as the current treatment paradigm and prognosis of this rare malignancy will be discussed based on a review of the literature.

## Case

**Fig. 1:**
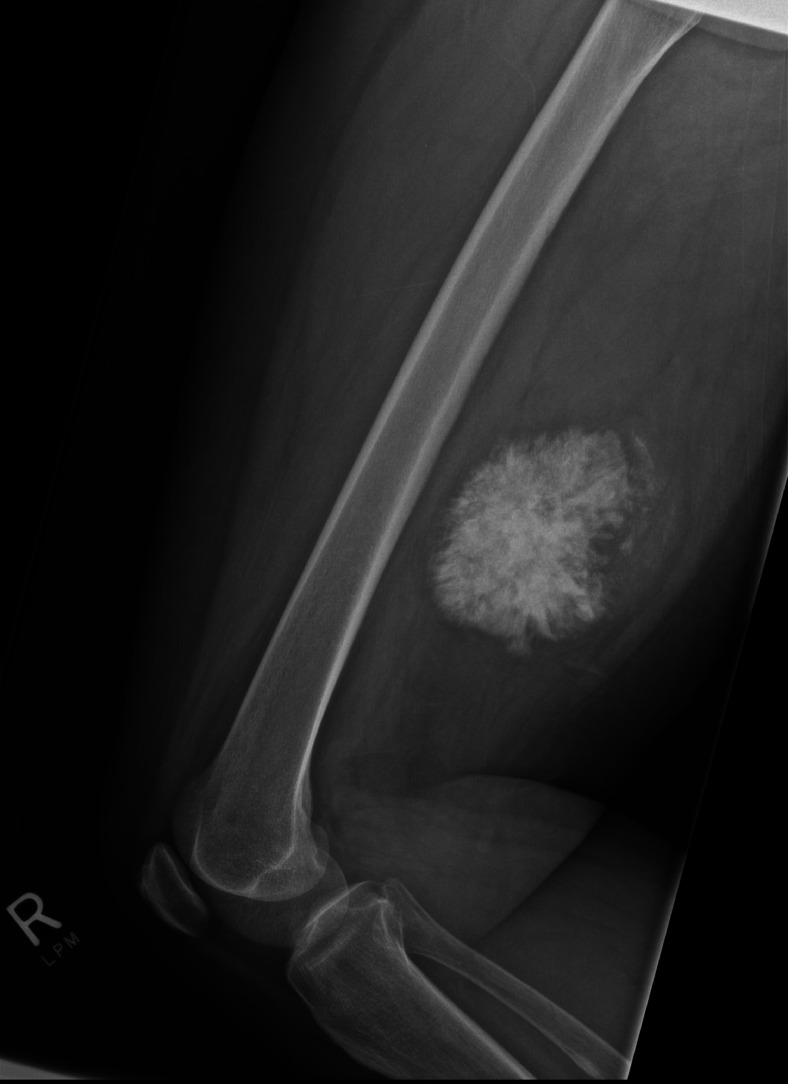
Right femur radiograph showed an extraskeletal calcified mass in the right posteromedial thigh with osteoid matrix. There are ill-defined central calcifications and margins. No involvement of the right femur. No periosteal reaction.

**Fig. 2:**
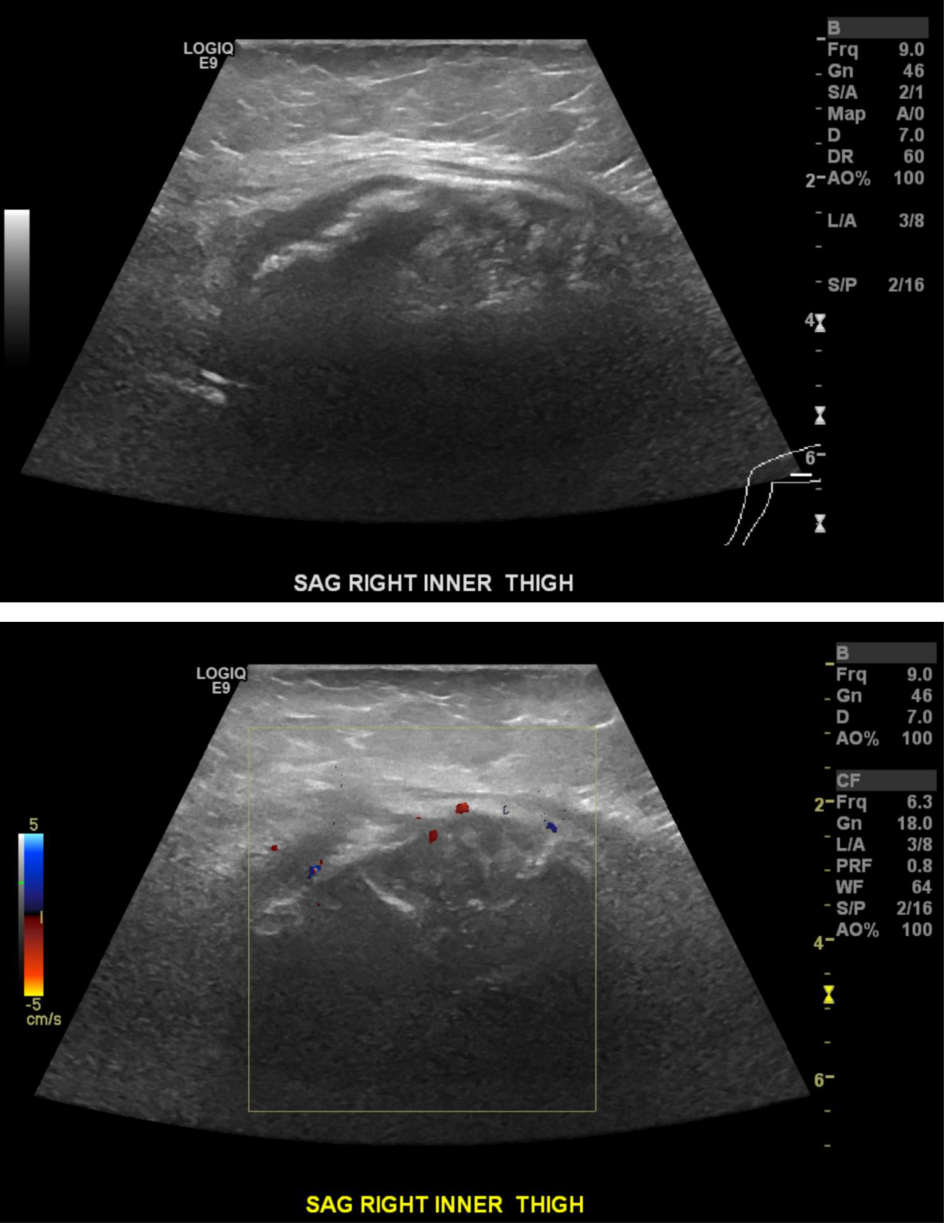
Targeted sonographic assessment of the right inner thigh showed a hypoechoic mass with dense calcification and significant posterior acoustic shadowing.

**Fig. 3:**
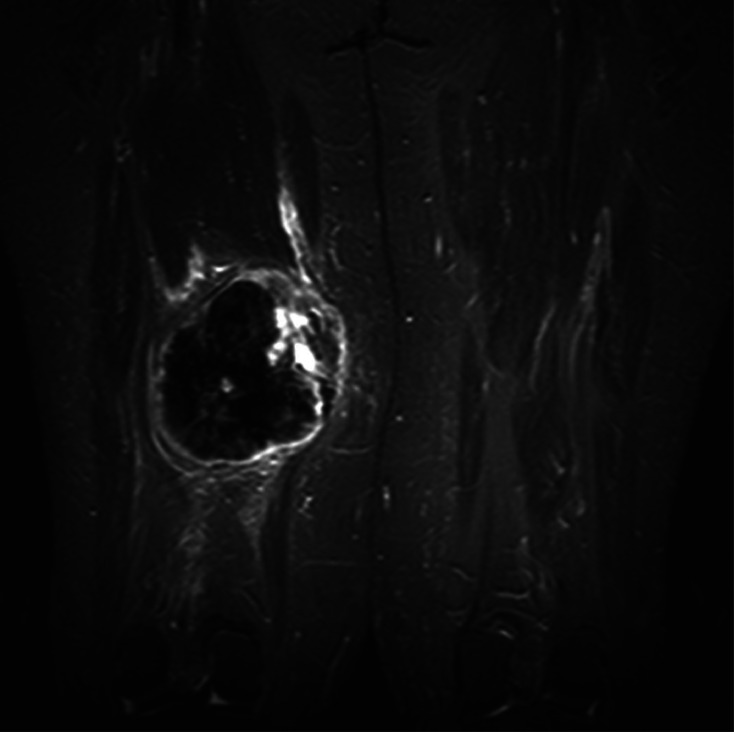

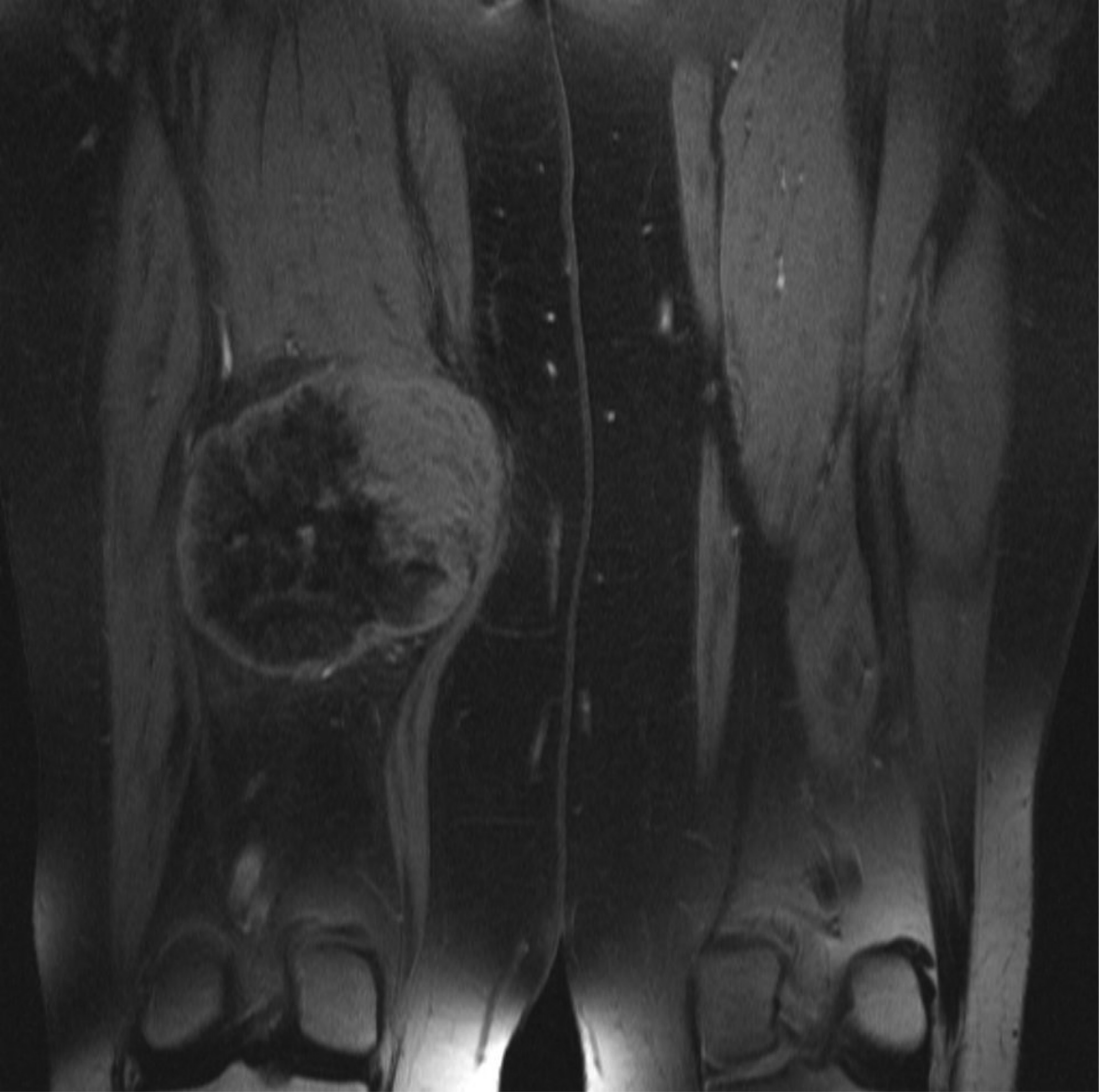

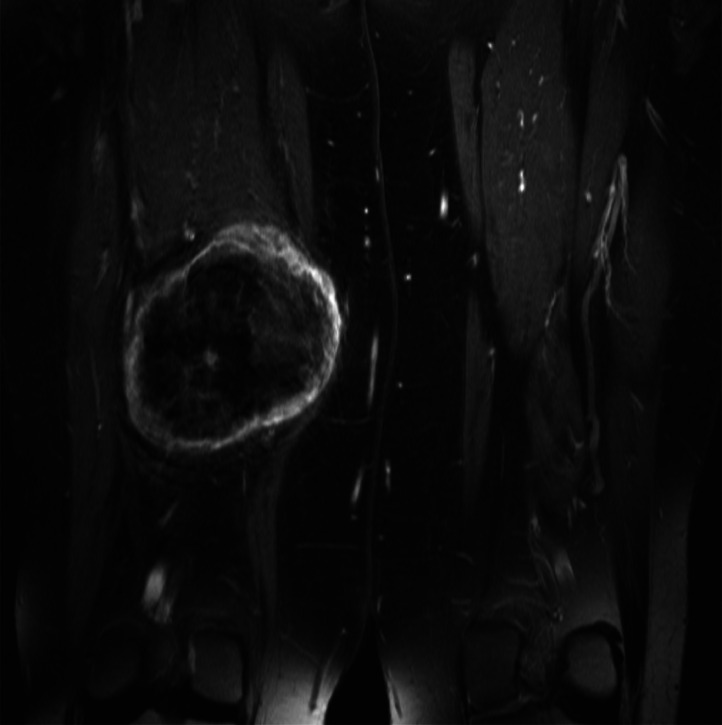
COR STIR, COR TSE T1 FS pregadolinium, and COR TSE T1 FS postgadolinium sequences demonstrate heterogeneously enhancing mass in the posteromedial compartment of the right thigh with eccentric soft-tissue and predominately peripheral enhancement with perilesional edema. Central low signal intensity reflects known matrix.

**Fig. 4:**
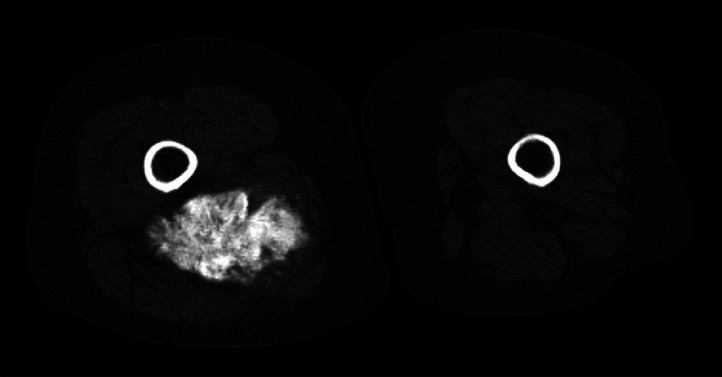
Axial unenhanced CT showing central calcification with ill-defined central mineralization and margins. No contiguity with the underlying femur. No periosteal reaction or aggressive features in the right femur.

**Fig. 5:**
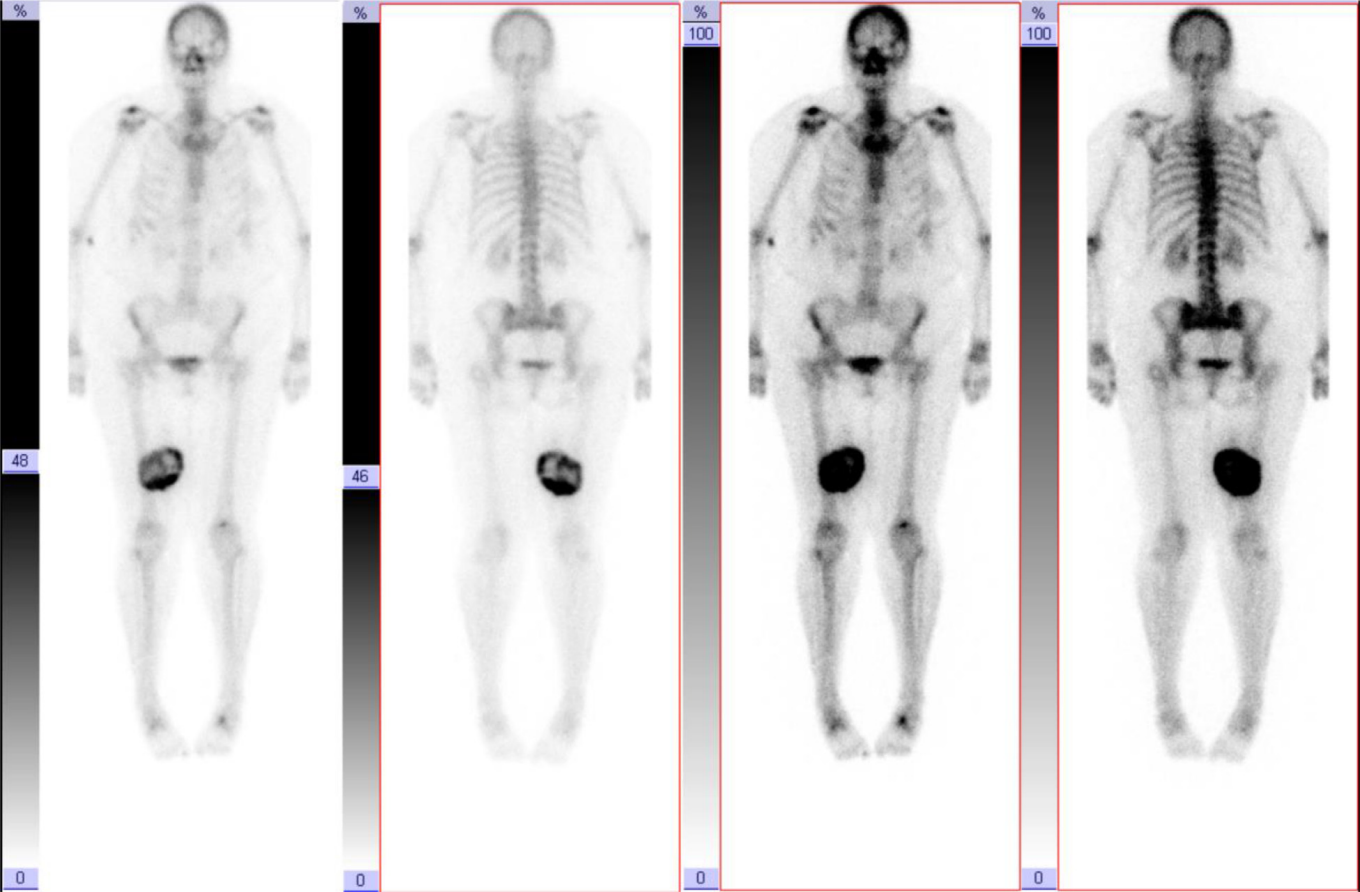
Bone scintigraphy showing increased uptake in the right posteromedial soft tissue mass in the thigh with greater uptake compared to the iliac crest. No suspicious uptake elsewhere.

**Fig. 6:**
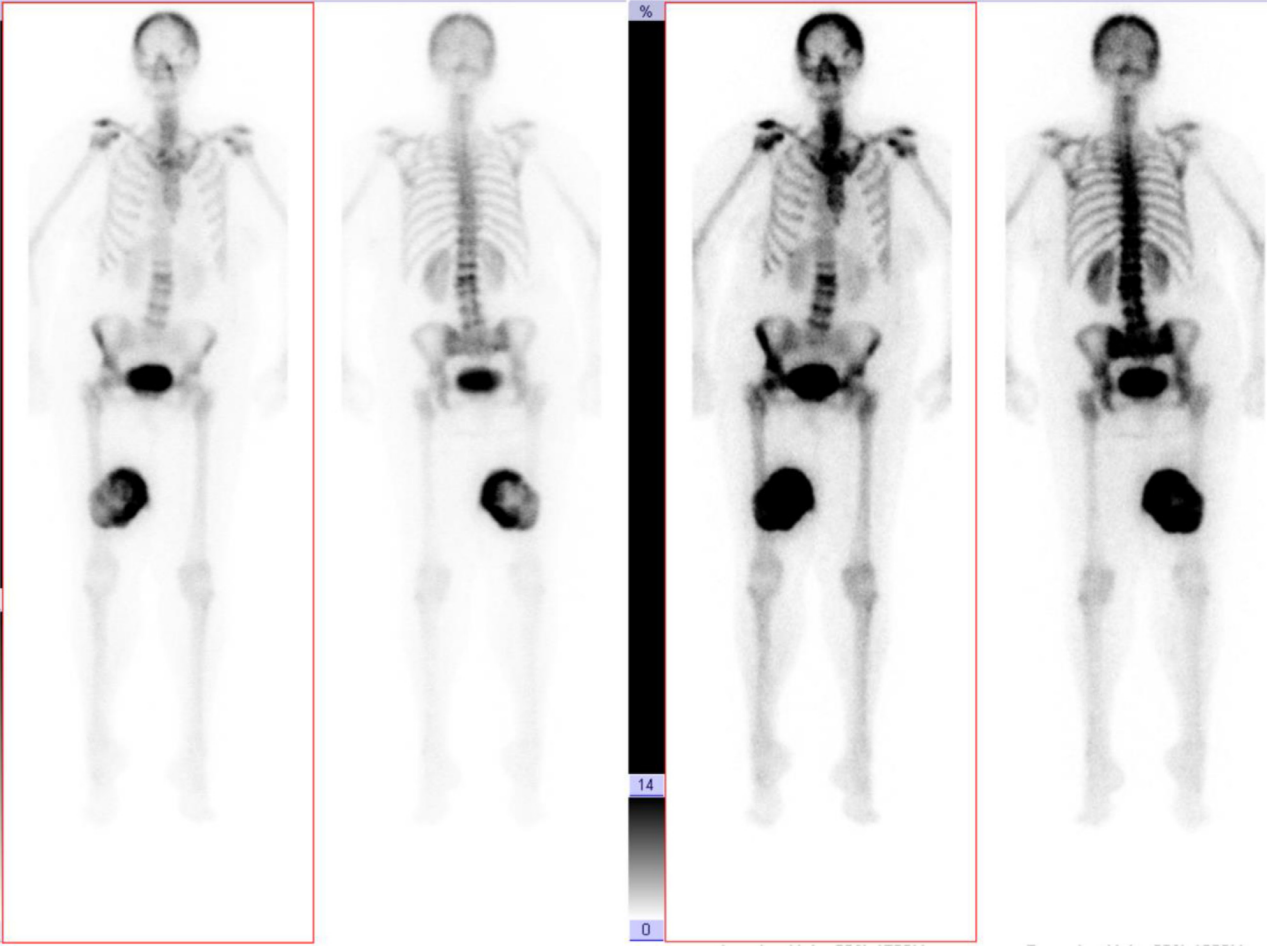
Six months repeat bone scintigraphy showing increased uptake in anterior iliac spines, right femur, and lumbar spine, consistent with metastatic disease.

A 67-year-old female presented with a 4-day history of a painless lump in her posterior right thigh. Radiographs of the right femur showed a calcified mass in the posteromedial soft tissues with ill-defined calcification centrally (Fig. 1). Targeted sonographic assessment of the right inner thigh showed a hypoechoic mass with dense calcification and significant posterior acoustic shadowing (Fig. 2). Magnetic resonance imaging without and with gadolinium contrast showed a 6.1 × 9.6 × 9.8 cm heterogeneously enhancing mass in the posteromedial aspect of the right thigh with infiltrative margins (Fig. 3). Axial unenhanced computed tomography (CT) showed central calcification with ill-defined mineralization and margins peripherally (Fig. 4). Bone scintigraphy showed an abnormally increased uptake in the mass (Fig. 5). The diagnosis of ESOS was favored based on the imaging findings, and this was confirmed via tissue sampling under Orthopedic guidance. CT staging performed thereafter showed the absence of metastatic disease in the chest, abdomen, and pelvis. The patient's case was discussed at the Sarcoma Multidisciplinary Tumor Board at the local hospital and the patient was started on chemotherapy regimen of ifosfamide (Ifex) and etoposide (Etopophos). However, after the second cycle of chemotherapy, the patient developed confusion and pancytopenia related to the chemotherapy and required IV antibiotics and pegfilgrastim (Neulasta). Furthermore, the patient developed atrial fibrillation and was consequently started on dabigatran (Pradaxa). The patient was hospitalized for recovery and pain control was challenging. Repeat bone scintigraphy 6 months after the initial work-up showed metastatic disease involving the axial and appendicular skeleton (Fig. 6) and as such, it was determined that the tumor is resistant to the primary chemotherapy agents. Second-line systemic therapy was thought to have an elevated risk of toxicity given recent history of atrial fibrillation and neutropenic sepsis. The patient's limited ambulation was another debilitating factor. Palliative radiation therapy was therefore elected, with intention of pain control, and was administered over 5 fractions. Physiotherapy was simultaneously offered to improve mobility.

## Discussion

ESOS is a relatively rare malignancy [11]. ESOS is recognized as being a high-grade malignant tumor, with a 90% recurrence (local or metastatic) rate anywhere within 3 years of excision [6,12]. Distal metastases are very common and are usually to the lungs, regional lymph nodes, and bone [10,11]. Patients with ESOS have an estimated 5-year disease-specific survival rate of 37%-47% [5,6,13]. A better prognosis is reported when the size of the tumor was less than 5 centimeters [14]. ESOS is more commonly found among patients aged 30 years and above and usually occurs in the lower extremities [12]. Additionally, because ESOS may mimic trauma in presentation (since both conditions usually present with tenderness and swelling), it is prone to misdiagnosis or delay [12].

The median age for ESOS is 47.5-61.0 and is slightly more common in men of that age range [15]. Tumors that have been analyzed microscopically appeared to have an infiltrative margin with occasional satellite nodules [16]. On radiographs, ESOS tumors were commonly seen as soft tissue opacities with mineralization of varying degrees. Furthermore, around half of ESOS cases develop calcification of the osteoid matrix in primary lesions [15].

On magnetic resonance imaging, ESOS tumors are typically seen as a well-circumscribed heterogeneous mass that is isointense to skeletal muscle on T1 and mildly hyperintense relative to skeletal muscle on T2. Hemorrhage is usually seen with hyperintense foci on both T1 and T2, and in extreme cases, the hemorrhage may mimic a hematoma [15].

It is crucial to distinguish ESOS from other, more common primary differential conditions such as myositis ossificans (MO) and extraskeletal chondrosarcoma. MO usually presents with a history of trauma and demonstrates progressive peripheral calcification producing a characteristic “zone phenomenon” on imaging. MO is a benign condition but if biopsied can end up resembling osteosarcoma on pathology [17,18]. As for extraskeletal chondrosarcoma, radiographic findings show chondroid matrix mineralization, described as having "ring-and-arc" morphology, compared to the osteoid matrix seen with osteosarcoma [19]. These features can be better appreciated with CT or magnetic resonance imaging.

The overall prognosis for ESOS is poor [15]. While surgical excision is the mainstay of treatment, neoadjuvant therapy (radiotherapy and chemotherapy) can help increase the surgical success rate and reduce the risk of recurrence. According to 2 review articles, radiation and chemotherapy were not associated with a lower incidence of death due to disease or a longer event-free survival [3,20]. However more recently, a large-scale study that included 266 ESOS patients presented by the European Musculoskeletal Oncology Society observed a higher survival rate in patients who received a perioperative chemotherapy regimen that included doxorubicin, ifosfamide and cisplatin [13]. Another study evaluating 43 patients also found that in patients who received chemotherapy, the relapse rate was lower in the platinum-based group (41%) as opposed to a nonplatinum-based group (100%; *P* = .02) [1]. Further standardized treatment protocols and larger scale studies are needed to outline the role of radiotherapy and chemotherapy in this disease.

Patients with ESOS may present with various symptoms and signs, depending on the stage of the tumor. Therefore, increasing familiarity with this disease is important for the complete assessment of ESOS patients and understanding the long-term prognosis while establishing consensus guidelines for the management of ESOS.

## Patient consent

Written, informed consent for publication of their case was obtained from the patient.
